# ID 163 - Avaliação do Relato do Envolvimento do Paciente e do Público nos Relatórios Técnicos no Brasil Publicados em 2023-2024

**DOI:** 10.5327/2237-9622.2025.v34s1.163

**Published:** 2025-11-25

**Authors:** Aléxia Gabriela da Silva Vieira, Aline Rocha, Ana Carolina Nunes Pinto, Amanda Alves Assis Garcia, Álvaro Nagib Atallah

**Keywords:** participação social, perspectiva do paciente, inclusão, relato da informação

## Abstract

**Introdução:**

Nos últimos anos, esforços têm sido feitos para melhorar metodologias e envolver pacientes em relatórios técnicos, mas a qualidade inconsistente dos relatos dificulta a compreensão de seu impacto. Nesse contexto, o objetivo deste resumo foi descrever as características do relato de envolvimento do paciente e do público no item perspectiva do paciente (PP) dos relatórios técnicos publicados pela Conitec, elencar os desafios para participação do público, fornecer sobre itens da ferramenta Guidance for Reporting Involvement of Patients and the Public Short Form (GRIPP2-SF) que poderiam ser incorporados nos relatórios técnicos.

**Método:**

Realizamos uma análise retrospectiva dos relatórios técnicos publicados pela Conitec publicados entre 6 de setembro de 2023 e 6 de setembro de 2024. Coletamos dados de caracterização dos relatórios que possuíam o item PP ou somente a consulta pública (CP), além dos desafios para participação social. Utilizamos o guia de relato GRIPP2-SF, que possui cinco itens e tem o objetivo de melhorar a qualidade do relato do envolvimento de pacientes e do público em pesquisas de saúde e assistência social em qualquer tipo de estudo, para fornecer de itens que poderiam ser incorporados aos relatórios técnicos. Realizamos uma análise descritiva dos dados, apresentando-os em porcentagem e média.

**Resultados:**

Setenta e um relatórios foram avaliados, sendo 7.371 participantes inscritos. Destes, quatro documentos não houve nenhuma participação social. Do total, 63% (45) possuíam o item PP e 37% (26) abordaram detalhes sobre a CP. Em média, o prazo da chamada pública para participação do item PP foi de 13 dias e a CP foi de 18 dias. O paciente era eleito para coleta das informações para o item PP por consenso entre os pacientes ou por sorteio, sendo então coletado seu relato de experiência e transcrito um resumo para o relatório técnico. Por outro lado, os relatórios que continham apenas o item CP utilizaram um formulário composto por duas partes: características do paciente e contribuição do paciente. Esta última, estruturada em três blocos de perguntas com o objetivo de conhecer a opinião do participante sobre a recomendação inicial da Conitec; a experiência prévia com a tecnologia em análise e a experiência prévia com outras tecnologias. Os resultados da CP foram apresentados em formato de resumo com as contribuições dos pacientes e comentários do grupo executor do relatório. Os desafios identificados para participação social foram: ausência de inscrição na chamada pública e não conformidade com os critérios de inclusão. Os itens do GRIPP2-SF que estavam descritos nos relatos foram: objetivos, métodos e resultados do estudo. Por outro lado, os itens ‘discussão e conclusões’ e ‘reflexões e perspectiva clínica’ poderiam ser incorporados para otimizar o aproveitamento da informação dos pacientes e fomentar a participação social.

**Conclusão:**

Os desafios para envolver pacientes na produção de relatórios técnicos refletem o panorama global, com a adesão às iniciativas e abordagens dinâmicas para extração de informações sendo frequentemente mencionadas na literatura, o que apoia nossos achados. Assim, é essencial aproveitar integralmente a informação do envolvimento do paciente para aumentar o engajamento e melhorar o relato. O guia GRIPP2-SF pode ser uma ferramenta útil nesse contexto, devido à sua flexibilidade e abordagem didática em relação aos itens desejáveis.

